# UNC0638 inhibits SARS-CoV-2 entry by blocking cathepsin L maturation

**DOI:** 10.1128/jvi.00741-25

**Published:** 2025-06-18

**Authors:** Yongjun Chen, Yujin Shi, Xiaoyan Zuo, Xiaojing Dong, Xia Xiao, Lan Chen, Zichun Xiang, Lili Ren, Zhuo Zhou, Wensheng Wei, Xiaobo Lei, Jianwei Wang

**Affiliations:** 1 NHC Key Laboratory of System Biology of Pathogens, Christophe Mérieux Laboratory National Institute of Pathogen Biology, Chinese Academy of Medical Sciences & Peking Union Medical College, Beijing, China; 2 Key Laboratory of Pathogen Infection Prevention and Control (Peking Union Medical College) Ministry of Education 127394, Beijing, China; 3 State Key Laboratory of Common Mechanism Research for Major Diseases, Suzhou Institute of Systems Medicine, Chinese Academy of Medical Sciences & Peking Union Medical College 570570, Suzhou, Jiangsu, China; 4 Biomedical Pioneering Innovation Center, Peking-Tsinghua Center for Life Sciences, Peking University Genome Editing Research Center, State Key Laboratory of Gene Function and Modulation Research, School of Life Sciences, Peking University 117978, Beijing, China; The Ohio State University, Columbus, Ohio, USA

**Keywords:** SARS-CoV-2, EHMT2, UNC0638, viral entry, cathepsin L

## Abstract

Since the outbreak of SARS-CoV-2, viral mutations have posed significant challenges in identifying therapeutic targets and developing broad-spectrum antiviral drugs. Post-translational modifications of genes involved in interferon production and signaling pathways play a crucial role in regulating interferon responses. In this study, we employed CRISPR-Cas9 screening based on adenine base editors to investigate functional amino acids in 1,278 innate immune-related genes. This approach, which converts A-T base pairs into G-C base pairs to probe the functional importance of specific amino acids, allowed us to identify 17 vital factors involved in SARS-CoV-2 infection. Among the candidate genes, genetic knockdown of EHMT2 exhibited the strongest antiviral effect. Further analysis revealed that UNC0638, a selective inhibitor of EHMT2, significantly reduced the endosomal entry of SARS-CoV-2 in pseudovirus assays. The observed inhibitory effect was consistently observed across multiple SARS-CoV-2 variants, including Alpha, Beta, Delta, and Omicron. Mechanistically, UNC0638 reduced mature cathepsin L (CTSL) levels, impairing the proteolytic cleavage of SARS-CoV-2 spike protein and subsequent membrane fusion, a critical step for viral entry. Our findings uncover EHMT2 as a host dependency factor and reveal the antiviral mechanism of EHMT2 inhibitors through CTSL maturation blockade. These results advance the understanding of host factors in SARS-CoV-2 infection and provide a strategic framework for developing host-targeted antiviral therapies.

**IMPORTANCE:**

In this study, we demonstrated that knockdown or knockout of EHMT2 inhibited SARS-CoV-2 infection, and inhibitors of EHMT2, including UNC0638, UNC0642, and BIX01294 showed similar restrictive effects. Mechanistically, the EHMT2 inhibitor UNC0638 restricts spike-mediated cell entry by inhibiting the maturation of CTSL, a critical protease required for SARS-CoV-2 entry via the endosomal pathway. Importantly, CTSL is not only essential for SARS-CoV-2 but also plays a key role in the entry of other coronaviruses that utilize similar pathways. Therefore, EHMT2 inhibitors could have broader applications as pan-coronavirus therapeutic agents.

## INTRODUCTION

Coronaviruses (CoV) are enveloped, positive-sense RNA viruses with genomes of approximately 26–32 kb long with a short untranslated region at both 5′ and 3′ terminal (1–3). The genome encodes four structural proteins: spike (S) protein, envelope protein, nucleocapsid (N) protein, and membrane (M) protein, along with 16 non-structural proteins (NSP1-16) and approximately eight accessory proteins (1–3). Seven coronaviruses are known to infect humans, including severe acute respiratory syndrome coronavirus (SARS-CoV), Middle East respiratory syndrome coronavirus (MERS-CoV), SARS-CoV-2, human coronavirus HKUI (HCoV-HKU1), HCoV-NL63, HCoV-OC43, and HCoV-229E (3). The first three are highly pathogenic and cause severe respiratory diseases, whereas the latter four typically result in mild, cold-like symptoms (4–6). Since its emergence in 2019, SARS-CoV-2 has caused an unprecedented public health crisis and enormous economic losses. While vaccines, monoclonal antibodies, and antiviral drugs have proven effective in preventing and treating infections, the continuous evolution of SARS-CoV-2 variants poses a growing challenge (7–10). Variants such as Delta, Omicron, and others have demonstrated the ability to partially or fully escape immune responses, making it difficult for previously effective vaccines and treatments to offer broad protection (9).

Recent studies have also identified mutations in key viral proteins, such as RNA-dependent RNA polymerase (RdRp) and main protease (Mpro), which confer resistance to clinically approved antiviral drugs, including remdesivir, nirmatrelvir, azvudine, and W116 (11–13). The efficacy of direct-acting antivirals has diminished, highlighting the urgent necessity for the discovery and development of host-targeting therapeutics to tackle the challenges presented by emerging resistant variants. To develop host-directed antivirals, several studies have focused on understanding the interactions between the virus and host cells. These investigations have identified numerous host factors that play important roles in the replication of SARS-CoV-2. For example, host factors such as transmembrane protein 41B (TMEM41B), transmembrane protein 106B (TMEM106B), sorting Nexin 27 (SNX27), and GATA-binding protein 6 (GATA6) have been found to promote viral replication, while lymphocyte antigen 6 complex locus E (LY6E), cholesterol 25-hydroxylase (CH25H), and phospholipid scramblase 1 (PLSCR1) inhibit viral replication (14–21). These host factors can serve as potential targets for the development of antiviral drugs.

Interferons are central to the host’s innate immune response against SARS-CoV-2 infection, particularly by regulating SARS-CoV-2 replication through key molecules involved in interferon production and downstream signaling pathways (22). Increasing evidence indicates that post-translational modifications (PTMs) are critical in regulating antiviral innate immune responses. These modifications, including phosphorylation, ubiquitination, and acetylation, play key roles in modulating the activity, stability, and interactions of innate-immune-related proteins (23–25). However, the role of PTMs of innate immune-related genes in SARS-CoV-2 infection is unclear. Considering the PTMs focused on lysine, serine, threonine, tyrosine, characteristics of the corresponding codon, and canonical CRISPR screening can’t accurately acquire the vital amino acids, we used the CRISPR-Cas-mediated base-editing technology to edit adenine base editor (ABE) for accurately probing functional amino acid residues of PTMs. The ABE mediated the precise conversion of A-T to G-C in genomic DNA with the assistance of single guide RNA (sgRNA) without introducing insertions or deletions, which can accurately account for the role of PTMs (26, 27).

In this study, we developed a CRISPR-Cas9-ABE-based screening strategy to profile the functional impact of PTM sites across 1,278 innate immune-related genes on SARS-CoV-2 infection. Our screen revealed significantly enriched sgRNAs targeting five key genes: TRIM28, SNX27, EHMT2, MAP2K1, and CIR, as well as control sgRNAs targeting ACE2. Notably, EHMT2 emerged as a top candidate, with five distinct target sites identified through sgRNA enrichment. Functional validation demonstrated that short interfering RNA (siRNA)-mediated knockdown of EHMT2 significantly reduced SARS-CoV-2 replication. Consistent with these genetic findings, three pharmacological inhibitors of EHMT2 (BIX01294, UNC0638, and UNC0642) exhibited dose-dependent antiviral activity against SARS-CoV-2. Mechanistically, all three inhibitors were found to reduce the level of mature CTSL, thereby impairing viral entry. These findings suggest that EHMT2 is a promising host factor for therapeutic intervention, and its inhibition exhibits potent antiviral activity against SARS-CoV-2.

## RESULTS

### Identification of host factors in SARS-CoV-2 infection using ABE-based CRISPR-Cas9 screening

**Fig 1:**
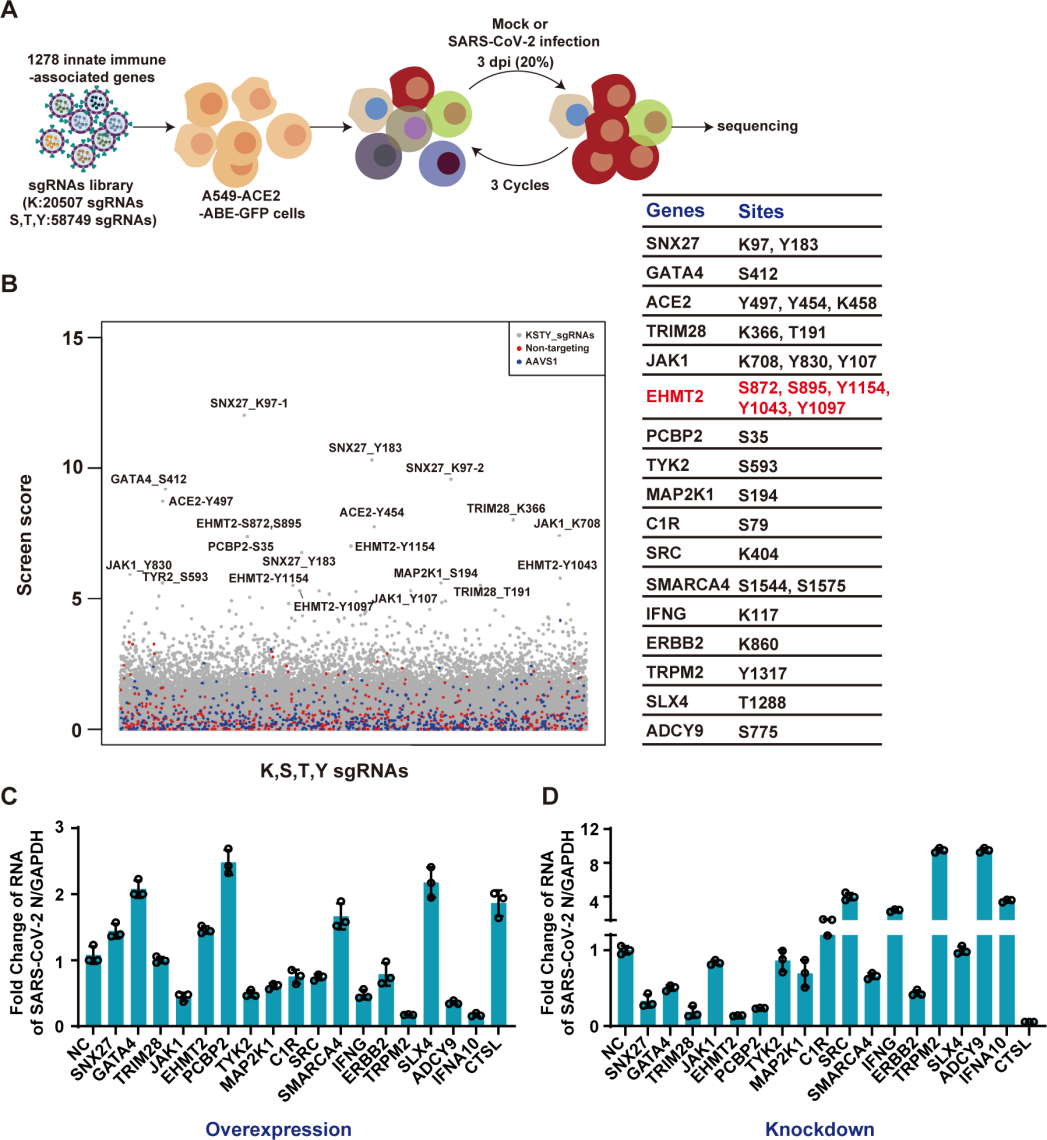
ABE system screens identify host factors for SARS-CoV-2 infection. (A) schematic diagram of the screening process. ACE2 and ABE-GFP expressing A549 cells transduced with a lentiviral sgRNA library targeting innate immune-related molecules. The cells were infected with SARS-CoV-2 at an MOI of 0.2. DNA was extracted from surviving cells after three rounds of infection and analyzed for sgRNA enrichment. (B) Gene enrichment from ABE screen of SARS-CoV-2 infection. (Left) Bubble plot showing data from SARS-CoV-2 screen. (Right) List of differentially enriched genes and their corresponding mutation sites. (C) Overexpression verification of candidate genes. Candidate genes were overexpressed in HEK 293T-ACE2, which were then infected with SARS-CoV-2 at an MOI of 0.1 for 24 h. Intracellular SARS-CoV-2 RNA levels were quantified with RT-qPCR, using GAPDH as an internal control. (D) Loss-of-function screen to identify functional host factors. Candidate genes were silenced using siRNA in A549-ACE2 cells, followed by infection with SARS-CoV-2 at an MOI of 0.1 for 24 h. Intracellular SARS-CoV-2 RNA levels were quantified by RT-qPCR, with GAPDH as the internal control.

Canonical CRISPR-Cas9 screening investigates gene function by introducing insertions or deletions to cause gene knockout (KO). In contrast, ABEs generate precise mutations by fusing an adenosine deaminase enzyme to a catalytically impaired Cas9 protein, enabling directed A-T to G-C base pair conversions in genomic DNA with the assistance of sgRNA (28, 29). Here, a sgRNA library of 80,285 sgRNAs across KSYT sites within 1,278 genes was transduced into A549-ACE2-ABE-GFP cells, and these cells were followed by puromycin selection for stable expression. The library contains 500 non-targeting sgRNAs and 499 sgRNAs editing AAVS1 as negative control and includes 30 sgRNAs targeting ACE2 as positive control. SgRNA-integrated cells were infected with SARS-CoV-2 at a multiplicity of infection (MOI) of 0.2 for 72 h. Human genes enriched in surviving cells were analyzed by the ZFCiBAR algorithm (Fig. 1A), leading to the identification of 34 sgRNAs targeting 17 genes (Fig. 1B). Among the enriched genes, ACE2 (viral entry receptor) and IFNG were prominently listed. Additionally, genes such as TRIM28 and SNX27, which have been recently implicated in SARS-CoV-2 infection, were markedly enriched, confirming the reliability of the screen.

To validate the potential candidate genes, we overexpressed each gene in cells and infected them with SARS-CoV-2 at an MOI of 0.1. CTSL was used as a positive control, and interferon alpha 10 served as a negative control. Overexpression of JAK1, TYK2, MAP2K1, IFNG, TRPM2, and ADCY9 significantly inhibited viral infection, whereas overexpression of SNX27, GATA4, EHMT2, PCBP2, SMARCA4, and SLX4 enhanced viral infection (Fig. 1C). To further characterize the functional roles of these genes, we generated knockdown cells using siRNA and infected them with SARS-CoV-2 at an MOI of 0.1 for 24 h. Knockdown assays revealed that SNX27, GATA4, TRIM28, EHMT2, PCBP2, and ERBB2 acted as pro-viral factors, whereas SRC, ADCY9, and TRPM2 functioned as anti-viral factors (Fig. 1D). Notably, knockdown of EHMT2 resulted in the most substantial reduction in SARS-CoV-2 RNA levels. Given its strong pro-viral effect, we selected EHMT2 for further investigation in subsequent experiments.

### EHMT2 is a proviral factor in SARS-CoV-2 infection

**Fig 2:**
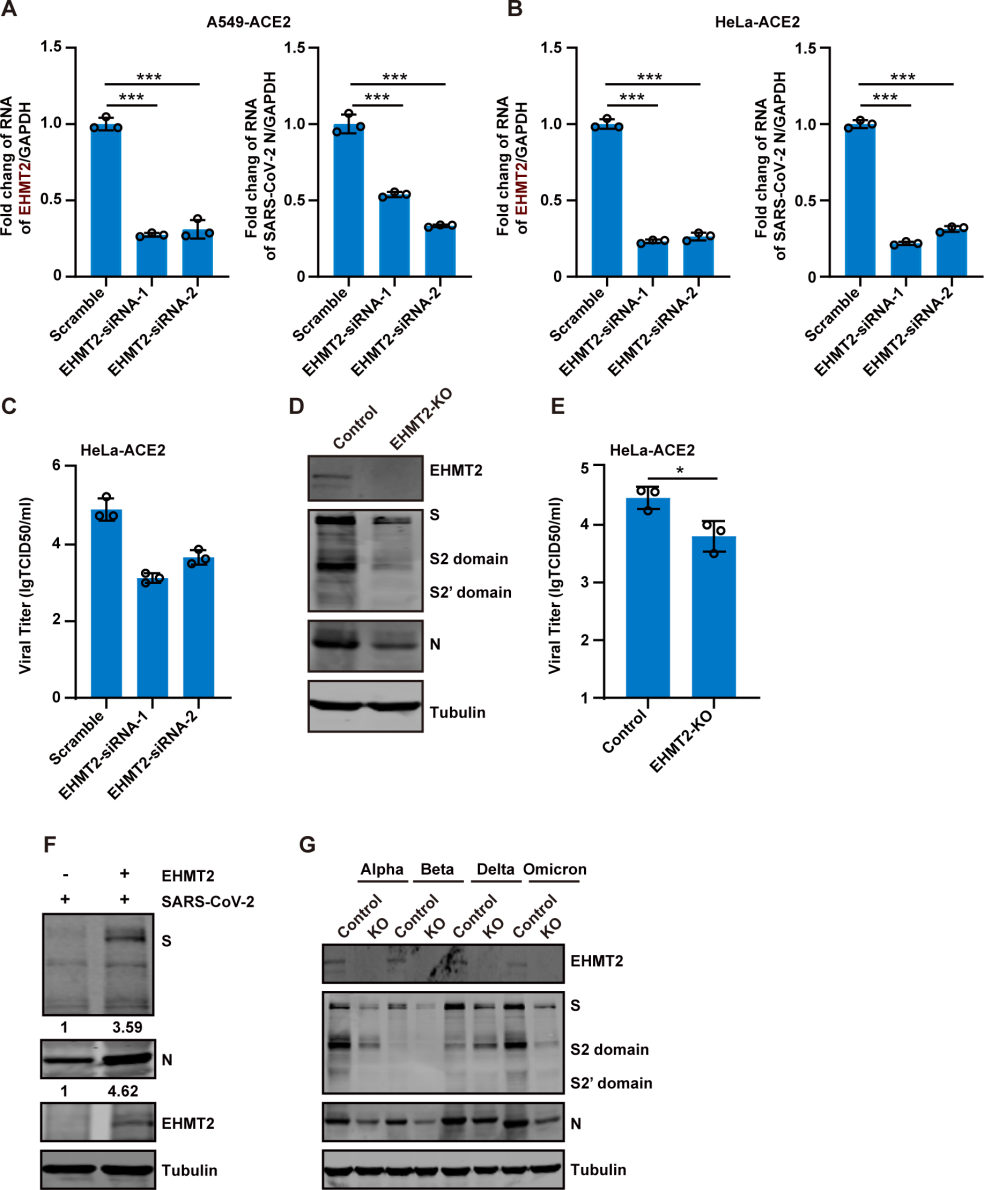
EHMT2 acts as a proviral factor in SARS-CoV-2 infection. (A and B) A549-ACE2 cells (**A**) and HeLa-ACE2 cells (**B**) were transfected with two independent siRNA targeting EHMT2, followed by infection with SARS-CoV-2 at an MOI of 0.1 for 24 h. Intracellular SARS-CoV-2 RNA levels were quantified by RT-qPCR, normalized to GAPDH. (C) Virus supernatants as in panel **B** were collected, and the virus titers were determined by TCID_50_ assay. (D) HeLa-ACE2 and HeLa-ACE2-EHMT2-KO cells were infected with SARS-CoV-2 at an MOI of 0.1 for 24 h. Cell lysates were collected and analyzed by western blot. (E) Virus supernatants as in panel **D** were collected, and the virus titers were determined by TCID_50_ assay. (F) HeLa-ACE2-EHMT2-KO cells were transfected with control plasmid or plasmid expressing EHMT2. After 24 h of transfection, cells were infected with SARS-CoV-2 at an MOI of 0.1 for 24 h. Cell lysates were evaluated using western blotting. (G) HeLa-ACE2-EHMT2-KO cells were infected with mutant strains of SARS-CoV-2 at an MOI of 0.1 for 24 h. All data are means ± SD. These experiments were repeated at least twice. ***P < 0.001, **P < 0.01, *P < 0.05, ns *P* > 0.05.

To further validate the role of EHMT2 in SARS-CoV-2 infection, we employed two distinct siRNAs targeting EHMT2 in A549-ACE2 and HeLa-ACE2 cells. Efficient knockdown of EHMT2 in A549-ACE2 cells was confirmed by real-time quantitative PCR (RT-qPCR), which showed a significant reduction in EHMT2 mRNA levels (Fig. 2A). This knockdown led to a marked inhibition of SARS-CoV-2 infection, as indicated by reduced viral RNA levels (Fig. 2A). Similar results were observed in HeLa-ACE2 cells (Fig. 2B). Furthermore, 50% tissue culture infectious dose (TCID_50_) assays demonstrated that the production of replicative viruses was significantly decreased in EHMT2 knockdown cells (Fig. 2C). These results suggest that the inhibitory effect of EHMT2 knockdown on SARS-CoV-2 infection is not cell type-specific.

To further assess its role, we generated an EHMT2-KO cell line and infected these cells with SARS-CoV-2 at an MOI of 0.1. Viral replication was significantly reduced in KO cells, as evidenced by decreased expression of the viral S and N proteins (Fig. 2D), and lower viral titers in TCID50 assays (Fig. 2E). Importantly, ectopic expression of EHMT2 in KO cells (HeLa-ACE2-EHMT2-KO) restored SARS-CoV-2 replication (Fig. 2F). To examine whether EHMT2’s proviral activity extends across SARS-CoV-2 variants, we infected KO cells with Alpha, Beta, Delta, and Omicron variants. All variants showed significantly reduced replication in EHMT2 KO cells (Fig. 2G), demonstrating the broad-spectrum role of EHMT2 in supporting SARS-CoV-2 replication.

### Antiviral activity of EHMT2 inhibitors

**Fig 3:**
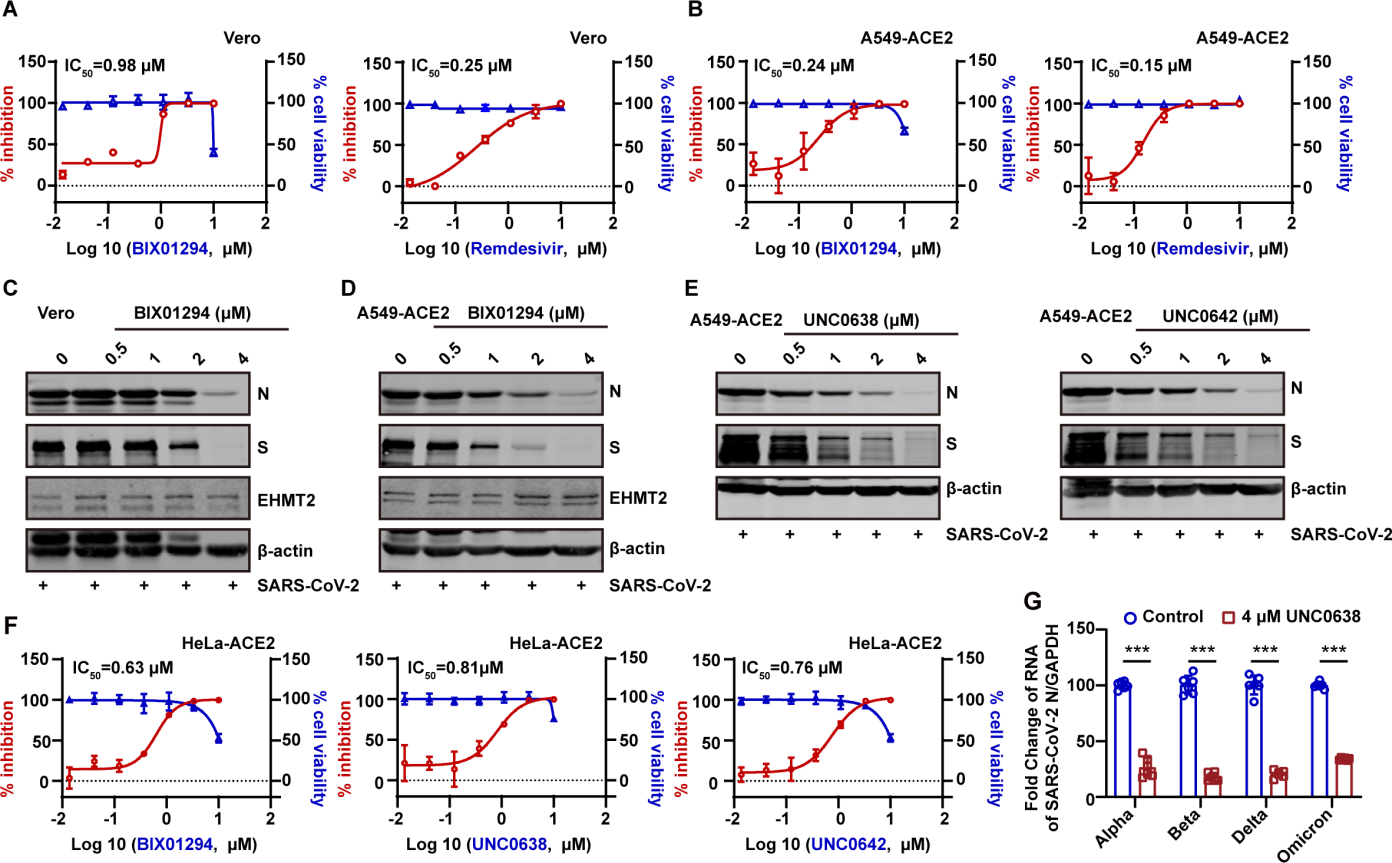
The antiviral activity of EHMT2 inhibitors. (A and B) Vero cells (**A**) and A549-ACE2 cells (**B**) were infected with SARS-CoV-2 at an MOI of 0.1 after treating with indicated doses of BIX01294 or Remdesivir. The SARS-CoV-2 RNA levels in supernatant were assessed through RT-qPCR. In parallel, their effects on cell viability were measured by CCK8 assay. The left *Y*-axis of the graphs represents % inhibition of the infection (red dots), and the right *Y*-axis of the graphs presents % cell viability (blue triangles). (C and D) Western blot detected the inhibition efficiency of BIX01294 in Vero cells (**C**) and A549-ACE2 cells (**D**). (E) Antiviral activity of EHMT2 inhibitors, UNC0638 (left) and UNC0642 (right), against SARS-CoV-2. A549-ACE2 cells were pretreated with inhibitors for 1 h and subsequently infected with SARS-CoV-2 at an MOI of 0.1 for 24 h. (F) % inhibition and % cell viability of BIX01294, UNC0638, and UNC0642 in HeLa-ACE2 cells. (G) Following either treatment or no treatment with UNC0638, HeLa-ACE2 cells were exposed to SARS-CoV-2 variants, including Alpha, Beta, Delta, and Omicron, at an MOI of 0.1 for 24 h. Intracellular SARS-CoV-2 RNA levels were quantified with RT-qPCR, using GAPDH as an internal control. Data are means ± SD. These experiments were repeated at least twice. ***P < 0.001, **P < 0.01, *P < 0.05, ns *P* > 0.05.

EHMT2 is highly expressed in various cancers, and several specific inhibitors have been developed as potential anticancer therapies (30–33). To evaluate the therapeutic potential of EHMT2 inhibition against SARS-CoV-2, we tested BIX01294, one of the earliest identified EHMT2 inhibitors. Pretreatment of Vero and A549-ACE2 cells with BIX01294 prior to SARS-CoV-2 infection resulted in dose-dependent antiviral activity, with IC_50_ values of 0.98 µM in Vero and 0.24 µM in A549-ACE2 (Fig. 3A and B). Notably, the inhibitor showed comparable efficacy to remdesivir (0.25 µM in Vero and 0.15 µM in A549-ACE2), a clinically approved direct-acting antiviral. The cytotoxic concentration was determined by assessing cell viability at similar concentrations (Fig. 3A and B). Western blot analysis confirmed that treatment of BIX01294 significantly reduced the expression of SARS-CoV-2 N and S proteins (Fig. 3C and D).

We further investigated two next-generation EHMT2 inhibitors, UNC0638 and UNC0642, which possess improved selectivity and potency compared to BIX01294 (34). Both compounds exhibited dose-dependent inhibition of SARS-CoV-2 infection in A549-ACE2 cells (Fig. 3E). Similar inhibitory effects were observed in HeLa-ACE2 cells, with IC_50_ values of 0.63 µM for BIX01294, 0.81 µM for UNC0638, and 0.76 µM for UNC0642 (Fig. 3F). Parallel cytotoxicity assays confirmed that UNC0638 exhibits low cytotoxicity. Moreover, previous reports have indicated that UNC0638 exhibits an improved cellular toxicity-to-function ratio compared to BIX01294 (34). Based on these pharmacological properties, UNC0638 was selected for further mechanistic investigation. Importantly, UNC0638 retained consistent antiviral activity across multiple SARS-CoV-2 variants, including Alpha, Beta, Delta, and Omicron (Fig. 3G), underscoring its broad-spectrum antiviral potential through EHMT2 inhibition.

### EHMT2 inhibitors exert antiviral activity independent of interferon signaling

**Fig 4:**
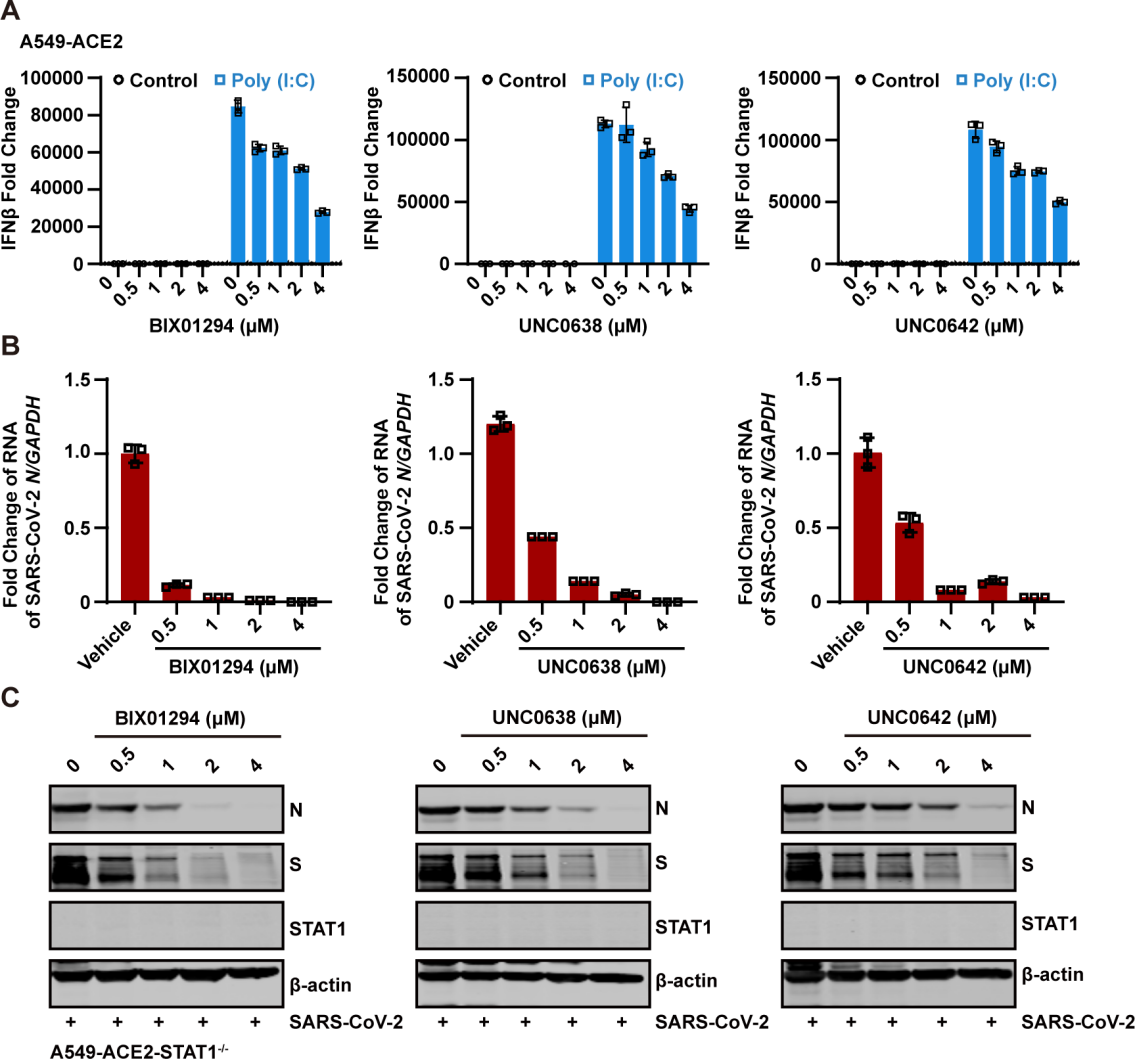
The antiviral activity of EHMT2 inhibitors is independent of the interferon pathway. (A) The effects of EHMT2 inhibitors, BIX01294 (left), UNC0638 (middle), and UNC0642 (right), on the interferon responses induced by high-molecular weight poly(I:C) were investigated. A549-ACE2 cells were transfected with poly(I:C) for 6 h after treating with varying concentrations of the inhibitors. The change of IFN-β was detected with RT-qPCR using GAPDH as an internal control. (B and C) A549-ACE2-STAT1^−/−^ cells were treated with EHMT2 inhibitors at indicated doses and then were infected with SARS-CoV-2 at an MOI of 0.1 for 24 h. Intracellular SARS-CoV-2 RNA levels were quantified with RT-qPCR, using GAPDH as the internal control (**B**). Intracellular SARS-CoV-2 protein levels were quantified with western blotting (**C**). Data are means ± SD. These experiments were repeated at least twice.

Previous studies have shown that depletion or inhibition of EHMT2 can enhance IFN responses to poly(I:C) stimulation in fetal bovine fibroblasts (35). To investigate whether the antiviral mechanism of EHMT2 inhibitors involves interferon signaling, we first assessed their effect on poly(I:C)-stimulated interferon responses in A549-ACE2 cells. Our results showed that poly(I:C) treatment induced IFNβ mRNA expression, and pretreatment with EHMT2 inhibitors (BIX01294, UNC0638, and UNC0642) did not potentiate this response (Fig. 4A). Rather, we observed a modest reduction in IFNβ mRNA levels (Fig. 4A), suggesting that their antiviral activity does not involve enhanced interferon production.

To definitively exclude the involvement of the interferon signaling, we generated STAT1-deficient A549-ACE2 cells (A549-ACE2-STAT1^−/−^), which are incapable of mounting type I interferon responses due to the absence of downstream signaling components. Following treatment with each EHMT2 inhibitor, the cells were infected with SARS-CoV-2 at an MOI of 0.1. Notably, all three EHMT2 inhibitors remained effective in suppressing SARS-CoV-2 replication in STAT1-deficient cells (Fig. 4B and C), further demonstrating that their antiviral activity is independent of the IFN signaling.

### UNC0638 targets an early post-entry step in the SARS-CoV-2 infection, primarily blocking the endosomal entry pathway

**Fig 5:**
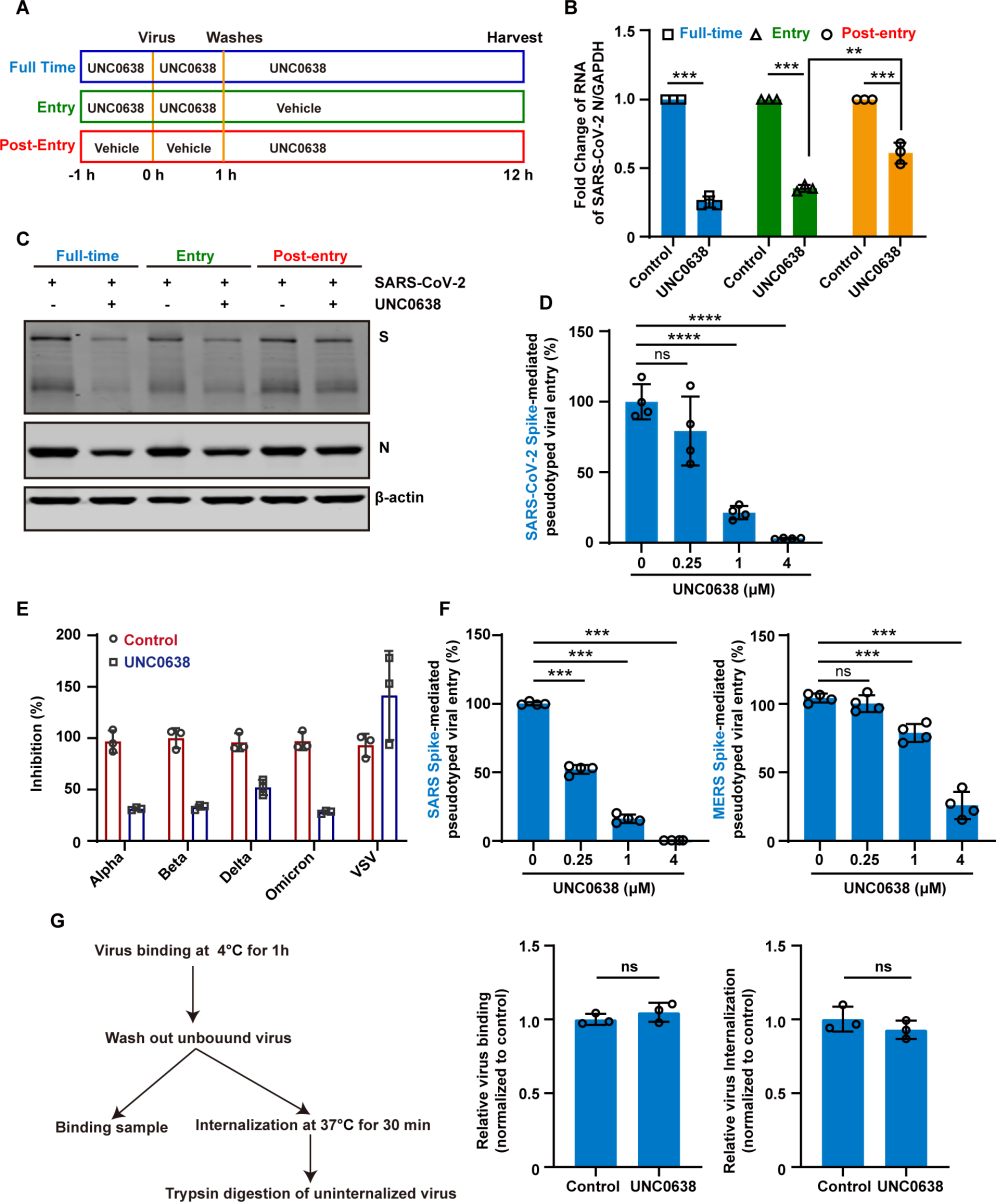
UNC0638 targets an early post-entry step primarily blocking endosomal entry of SARS-CoV-2. (A, B, and C) Schematic diagram of the time-of-drug-addition assay (A). HeLa-ACE2 cells were treated with UNC0638 at the different stages of the viral life cycle and categorized into full-time, entry, and post-entry groups. These cells were infected with SARS-CoV-2 at an MOI of 0.1 for 12 h. Intracellular SARS-CoV-2 RNA levels were quantified with RT-qPCR, using GAPDH as an internal control (**B**). Intracellular SARS-CoV-2 protein levels were quantified with western blotting (**C**). (D) Following the treatment with UNC0638, HeLa-ACE2 cells were infected with pseudovirions, and luciferase activity was assessed after 48 h post-infection. (E) After the treatment with UNC0638, HeLa-ACE2 cells were infected with pseudovirions that carry the S protein from various strains of SARS-CoV-2, including Alpha, Beta, Delta, and Omicron variants. The luciferase activity was assessed after 48 h post-infection. (F) Following UNC0638 treatment, HeLa-ACE2 (left) or HeLa-DPP4 (right) cells were infected with SARS-CoV-1 or MERS-CoV pseudovirion, respectively. Luciferase activity was assessed after 48 h post-infection. (G) Viral binding and internalization in HeLa-ACE2 cells with or without UNC0638 treatment. Intracellular SARS-CoV-2 RNA levels were quantified with RT-qPCR, using GAPDH as an internal control. Data are means ± SD. These experiments were repeated at least twice. ***P < 0.001, **P < 0.01, *P < 0.05, ns *P* > 0.05.

To identify the stage of the SARS-CoV-2 life cycle targeted by EHMT2 inhibition, we performed a time-of-drug-addition assay during a single viral replication cycle (36), as illustrated in Fig. 5A. UNC0638 effectively inhibited viral infection when present throughout the entire infection period or during pretreatment (Fig. 5B and C). While post-infection addition, the antiviral activity was weaker compared to continuous treatment during the infection. These findings suggest that UNC0638 primarily targets the viral entry stage. To further investigate whether UNC0638 influences virus entry, we employed a vesicular stomatitis virus (VSV)-based pseudotyped virus production system. As shown in Fig. 5D, UNC0638 treatment significantly reduced infection by SARS-CoV-2 S-mediated pseudovirions in a dose-dependent manner. This inhibitory effect was observed not only with the original strain but also for multiple variants of concern, including Alpha, Beta, Delta, and Omicron (Fig. 5E). To further explore whether the inhibitory effect of EHMT2 blockade extends beyond SARS-CoV-2, we generated pseudoviruses bearing the S proteins of SARS-CoV and MERS-CoV. Treatment with UNC0638 markedly suppressed the entry of both SARS-CoV and MERS-CoV S-mediated pseudoviruses, indicating that EHMT2 inhibition confers broad-spectrum antiviral activity against human coronaviruses (Fig. 5F).

To evaluate the mechanism of UNC0638’s antiviral activity against SARS-CoV-2 infection, we systematically examined its effects on SARS-CoV-2 binding and internalization using the experimental workflow outlined in Fig. 5G. The result indicated that UNC0638 treatment had no significant impact on either (i) the initial attachment of SARS-CoV-2 to cell surfaces or (ii) subsequent viral internalization (assessed by trypsin resistance assay following 30 min incubation at 37°C) (Fig. 5G). These findings indicate that UNC0638 mediated its antiviral effects through post-internalization mechanism.

### UNC0638 suppresses SARS-CoV-2 endocytosis by inhibiting CTSL maturation

**Fig 6:**
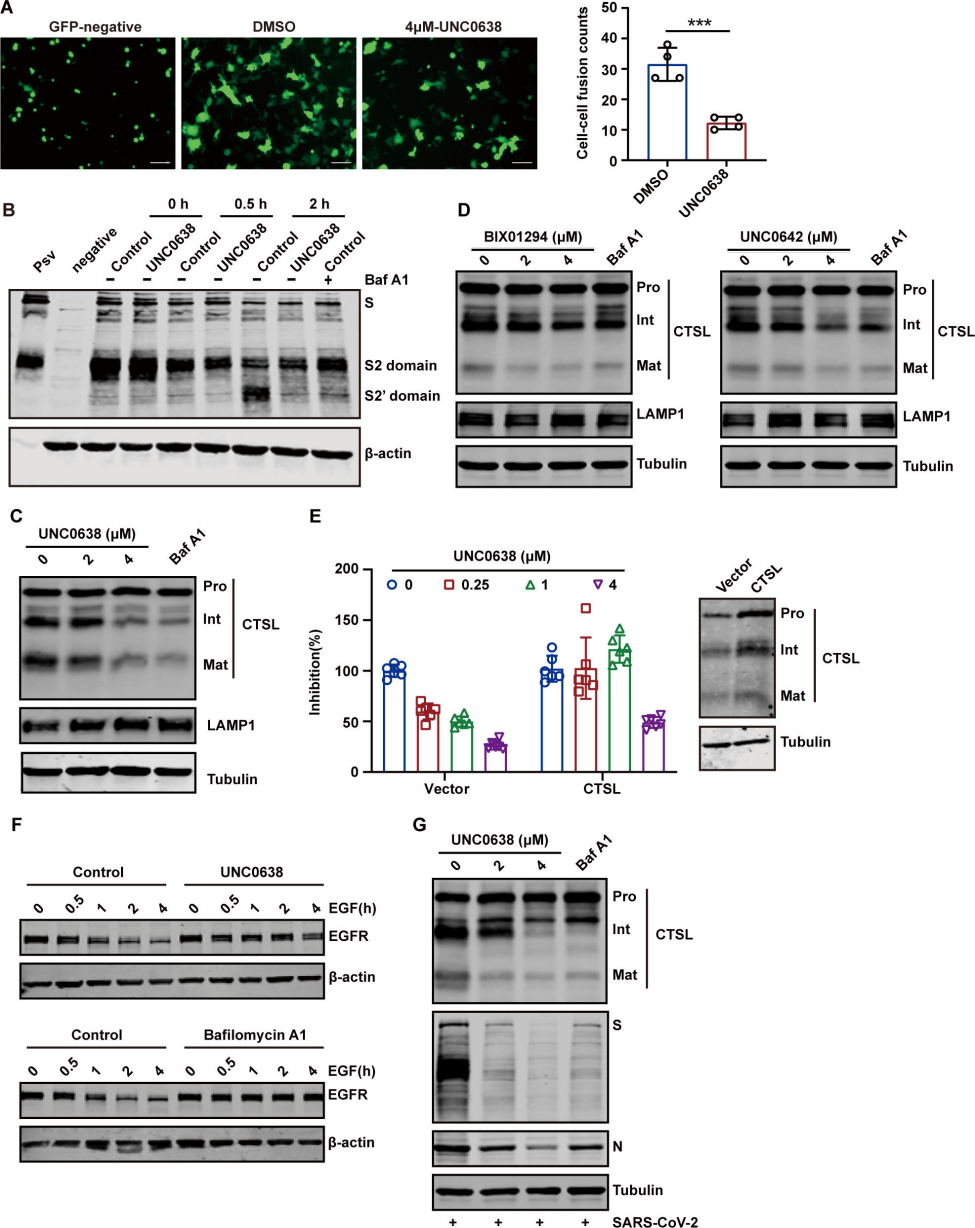
UNC0638 suppresses SARS-CoV-2 endocytosis by inhibiting the maturation of CTSL. (A) Left, representative images showing syncytia formation after incubating UNC0638-treated or UNC0638-untreated HeLa-ACE2 with HEK 293T expressing SARS-CoV-2 S and EGFP. Scale bars, 100 µm. Right, quantitative analysis of syncytia with ImageJ. (B) The cleavage of the SARS-CoV-2 S protein was examined in HeLa-ACE2 cells, both treated and untreated with UNC0638, following infection with pseudovirions over specified time intervals. Cell treated with bafilomycin A1 (Baf A1) (200 nM) served as a negative control. The level of S2′ domain was detected by western blotting. (C) Western blot analysis of CTSL protein levels in HeLa-ACE2 cells with or without UNC0638 treatment. Cell treated with Baf A1 (200 nM) served as a negative control. (D) The effects of BIX0194 and UNC0642 on CTSL levels in HeLa-ACE2 cells. (E) HeLa-ACE2 cells were transfected with control plasmid or plasmid expressing CTSL. After 24 h of transfection, cells were infected with pseudovirions after treatment with indicated doses of UNC0638. Left, luciferase activity was assessed after 48 hours post-infection. Right, detection of expression efficiency of CTSL in HeLa-ACE2 with western blotting. (F) HeLa-ACE2 cells were stimulated with 40 ng/mL of EGF for different time after treatment with UNC0638. Cells treated with bafilomycin A1 (200 nM) served as a negative control. The level of EGFR was detected by western blotting. (G) HeLa-ACE2 cells were pretreated with UNC0638 and infected with SARS-CoV-2 at an MOI of 0.1. Intracellular SARS-CoV-2 S, N, and CTSL protein levels were evaluated using western blotting. Data are means ± SD. These experiments were repeated at least twice. ***P < 0.001, **P < 0.01, *P < 0.05, ns *P* > 0.05.

Having established that UNC0638 does not affect viral binding or internalization, we sought to determine its mechanism of viral entry inhibition. We first examined whether UNC0638 interferes with S-mediated membranefusion using a cell-cell fusion assay. When HEK 293T cells transiently co-expressing SARS-CoV-2 S protein and EGFP (enhanced green fluorescent protein) were co-cultured with ACE-2-expressing HeLa cells, extensive syncytia formation was observed (Fig. 6A). Notably, UNC0638 treatment significantly reduced the S-mediated cell fusion (Fig. 6A). To further evaluate the impact of UNC0638 on SARS-CoV-2 S protein processing, we performed a pseudovirion infection assay. HeLa-ACE2 cells pretreated with UNC0638 were incubated with pseudovirion at 4°C for 1 h and then shifted to 37°C for the indicated times. The results demonstrated that S2′ cleavage, which was detectable 2 h post-infection, was significantly suppressed by UNC0638 treatment (Fig. 6B). As a control, cells treated with bafilomycin A1, an inhibitor of endosomal acidification, also suppressed S2′ cleavage.

During the cathepsin-dependent endosomal fusion pathway, ACE2 engagement exposes the S2′ cleavage site, facilitating the cleavage of the S2 subunit by CTSL (37). To investigate whether UNC0638 affects S2′ cleavage by interfering with CTSL maturation, we conducted western blot analyses. As shown in Fig. 6C, UNC0638 significantly decreased the levels of mature CTSL without affecting LAMP1 expression, indicating a specific effect on CTSL maturation. Similar results were observed in BIX01294- and UNC0642-treated group (Fig. 6D). To confirm the role of CTSL in the inhibitory effect of UNC0638, we overexpressed CTSL in HeLa-ACE2 cells and evaluated pseudovirion infection in the presence of UNC0638. The results showed that CTSL overexpression partially rescued pseudovirion infection, particularly at low concentrations of UNC0638 (0.25 µM and 1 µM) (Fig. 6E). To further validate the inhibitory effect of UNC0638 on the enzymatic activity of cathepsins, we additionally examined its impact on epidermal growth factor receptor (EGFR) degradation. Following activation by EGF, EGFR undergoes degradation mediated by cathepsins (38). As shown in Fig. 6F, EGF stimulation led to EGFR degradation, as expected. However, treatment with UNC0638 significantly resisted EGF-induced EGFR degradation, indicating that UNC0638 disrupts cathepsins activity. Bafilomycin A1, an inhibitor of endosomal acidification, was used as a control and showed a similar effect (Fig. 6F).

**Fig 7:**
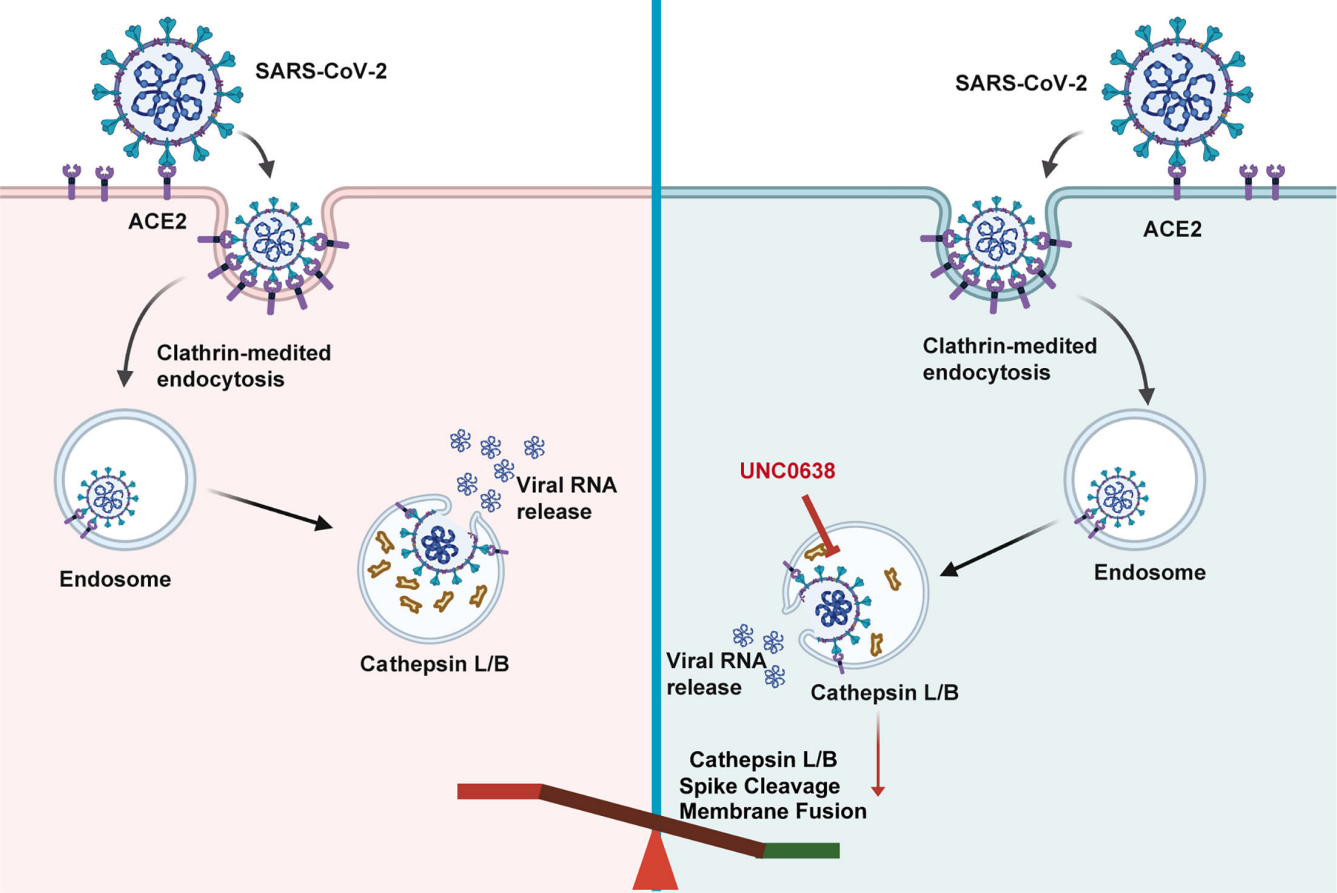
Schematic diagram illustrating the proposed mechanism by which UNC0638 antagonizes SARS-CoV-2 infection.

Lastly, to confirm the biological relevance of our pseudovirus findings, we infected HeLa-ACE2 cells with authentic SARS-CoV-2. Consistent with our pseudotyped virus results, UNC0638 treatment effectively inhibits CTSL maturation during SARS-CoV-2 infection (Fig. 6G). Collectively, these findings demonstrate that UNC0638 inhibits SARS-CoV-2 entry by suppressing CTSL-mediated cleavage of the S protein (Fig. 7).

## DISCUSSION

Type I IFNs play a crucial role in antiviral defense against SARS-CoV-2 (39–42). As an RNA virus, SARS-CoV-2 activates host intracellular pattern recognition receptors, such as the RIG-I signaling pathway, to induce IFN responses. Additionally, SARS-CoV-2 infection triggers the release of host DNA into the cytoplasm, where it is recognized by cyclic GMP-AMP synthase (cGAS), further amplifying IFN production (43). IFNs mediate their antiviral effects by upregulating the expression of hundreds of ISGs. However, the specific contributions of these innate immunity-associated genes in SARS-CoV-2 replication remain incompletely characterized. In our study, we conducted a CRISPR-Cas9 screening targeting 1,278 innate immunity-associated genes, incorporating KSTY amino acid mutations to disrupt potential post-translation modification sites. This screen identified 17 candidate genes, of which six genes exhibited pro-viral and three genes displayed anti-viral effects upon siRNA-mediated knockdown or plasmid-mediated overexpression. Given the high inhibitory efficiency observed after siRNA knockdown, EHMT2 was prioritized for further investigation. This observation aligns with a recent report by Sakai M. et al. (44), who conducted a systematic genome-wide CRISPR-Cas9 screen and identified EHMT2 as a pro-viral host factor essential for SARS-CoV-2 replication through a complex regulatory mechanism.

Our study demonstrates that UNC0638, an EHMT2 inhibitor, potently suppresses SARS-CoV-2 entry by impairing S protein cleavage and virus-endosome fusion, while having no effect on viral attachment or internalization. These findings indicate that UNC0638 targets the post-internalization, lysosome-dependent stage of viral entry. In the endocytic entry pathway, S protein cleavage is primarily mediated by lysosomal cysteine proteases CTSL and to a lesser degree by CTSB, which are crucial for viral membrane fusion and subsequent release of the viral RNA genome into the host cytoplasm (45, 46). The clinical relevance of this pathway is underscored by a recent study demonstrating a positive correlation between circulating CTSL levels and COVID-19 disease severity (47), highlighting the potential of CTSL as an attractive therapeutic target for SARS-CoV-2 infection. In this study, we identified UNC0638 as a novel inhibitor of CTSL activation with broad-spectrum antiviral activity against coronavirus infections.

Our analysis revealed that UNC0638 treatment significantly reduced the maturation of CTSL. Overexpression of CTSL counteracted the inhibitory effect of UNC0638 on S-mediated pseudovirion entry, supporting a functional link. Consistent with lysosomal dysfunction, UNC0638 also impaired EGF-induced EGFR degradation in HeLa-ACE2, a process known to depend on cathepsins activity. Together, these three lines of evidence: (i) inhibited CTSL maturation, (ii) CTSL-dependent rescue of viral entry, and (iii) impaired EGFR degradation, collectively demonstrate that UNC0638 disrupts lysosomal protease function. While these results clearly establish UNC0638’s effect on CTSL function, the molecular mechanisms remain to be fully elucidated. Several potential mechanisms could underlie this effect: (i) UNC0638 may directly inhibit CTSL protease activity by binding to the enzyme. (ii) It could disrupt lysosomal acidification. Lysosomal proteases, including CTSL, require an acidic environment for activation and maturation. (iii) UNC0638 may alter cholesterol distribution in lysosomal membranes, potentially affecting protease maturation and membrane fusion events. Notably, while several small-molecule CTSL/CTSB inhibitors have been reported (46, 48–51), our study is the first to implicate EHMT2 inhibition in CTSL maturation. This finding suggests an unexpected connection between histone methylation and lysosomal protease regulation that merits further exploration.

Although our mutational screening was originally designed to investigate PTM effects, we unexpectedly found that these mutations in EHMT2 primarily decreased protein expression, suggesting their impact may not be mediated through PTM alterations. This may explain why our results align with genome-wide CRISPR-Cas9 screening data, which identified similar host factors as regulators of SARS-CoV-2 infection (44). Overall, our study demonstrates that EHMT2 inhibition significantly suppresses SARS-CoV-2 replication and, for the first time, elucidates their novel mechanism of action through inhibiting CTSL activation. These findings provide valuable insights for future antiviral drug development.

## MATERIALS AND METHODS

### Cell lines and virus

The HEK 293T (embryonic kidney), A549 (human alveolar basal epithelial carcinoma), and HeLa (human cervical adenocarcinoma) cell lines were purchased from ATCC. To generate A549-ACE2, HeLa-ACE2, and HEK 293T-ACE2 cell lines, human ACE2 was expressed in the respective parental cell lines (21, 43). The HeLa-ACE2-EHMT2-KO cells were established through CRISPR-Cas9 technology. The sgRNA sequences used were “AGAAGTGACCCTGACGAAAG.” All cells were cultured in Dulbecco’s modified Eagle’s medium (Gibco), supplemented with 10% fetal bovine serum (Corning), and incubated at 37°C in a humidified atmosphere containing 5% CO_2_.

The SARS-CoV-2 virus used in this study originated from the previous isolation of our laboratory. All experiments involving live SARS-CoV-2 were conducted under biosafety level 3 conditions, following institutional and national biosafety guidelines.

### Antibody and reagents

The following antibodies were used in this study: rabbit polyclonal anti-SARS-CoV-2 S1 antibody (40150-T62), rabbit polyclonal anti-SARS-CoV-2 S2 antibody (40590-T62), and rabbit monoclonal anti-SARS-CoV-2 N protein antibody (40143-R109), all purchased from Sino Biological Inc. Mouse monoclonal anti-actin antibody (A5441) was from Sigma-Aldrich. Mouse polyclonal anti-α-tubulin antibody (11224-1-AP) was from Proteintech. Rabbit monoclonal anti-EGFR antibody (4267), rabbit monoclonal anti-LAMP1 antibody (9090), and rabbit polyclonal anti-STAT1 antibody (9172) were from Cell Signaling Technology. Rabbit monoclonal anti-EHMT2 antibody (ab185050) was from Abcam. Goat polyclone anti-CTSL (AF952-SP) was from R&D Systems.

EHMT2 inhibitor BIX01294 (T1959), UNC0638 (T3257), UNC0642 (T4166), and remdesivir (T7766) were purchased from TargetMol. Bafilomycin A1 (HY-100558) and EGF (HY-P7109) were purchased from MedChemExpress (MCE).

### ABE screening in A549-ACE2 cells

The ABE screening protocol was adapted from a previously described method (26). A549-ACE2 cells were transduced with a lentivirus expressing ABE-GFP and enriched by flow cytometry sorting. The ABE-GFP-expressing A549-ACE2 cells were subsequently infected with lentiviruses containing a sgRNA library targeting 1,278 genes. After 24 hours, the cells were treated with 1 µg/mL puromycin for five days to select for transduced cells. Approximately 2 × 10^7^ surviving cells were infected with SARS-CoV-2 at an MOI of 0.2. After 72 h, surviving cells were collected, cultured, and subjected to two additional rounds of viral infection and selection. Genomic DNA was extracted from the final surviving cell population using the TIAamp Genomic DNA Kit (TIANGEN, DP304). sgRNA sequences were amplified using KOD One PCR Master Mix (TOYOBO LIFE SCIENCE, KMM-101) and sequenced on an Illumina platform. Gene enrichment analysis and ranking were performed using ZFC^iBAR^ algorithm.

### CCK8 assay

Cells were seeded in 96-well plates at a density of 2 × 10^4^ cells per well. The following day, cells were treated with EHMT2 inhibitors (UNC0638, UNC0642, BIX01294) at indicated concentrations for 1 h at 37°C. After treatment, cell viability was assessed using the CCK-8 assay (Yeasen, 40203ES80) according to the manufacturer’s protocol. Briefly, 10 µL of CCK8 solution was added to each well, followed by incubation at 37°C for 2 h. Absorbance was measured at 450 nm using a microplate reader to assess cell viability.

### Real-time quantitative PCR

Total RNA was extracted using TRIzol reagent (Thermo Fisher Scientific, 15596018CN) according to the manufacturer’s instructions. For cDNA synthesis, 1 µg of total RNA was reverse transcribed using M-MLV Reverse Transcriptase (Promega, M1705) with oligo (dT) primers. The resulting cDNA was diluted 1:5 and used as a template for real-time quantitative PCR (RT-qPCR) using TB Green Premix Ex Taq II (TaKaRa, RR820A). The relative mRNA expression levels were calculated using the 2^−△△ct^ method with glyceraldehyde-3-phosphate dehydrogenase (GAPDH) as the reference gene.

### Absolute quantitative PCR for viral N mRNA

A549-ACE2 or HeLa-ACE2 cells were treated with indicated drugs, followed by SARS-CoV-2 infection. Supernatants were collected and subjected to RNA extraction, using Direct-zol RNA MiniPrep Kit (ZYMO RESEARCH, CA, USA) according to the manufacturer’s instructions. Viral copy numbers were measured by RT-PCR using primers and probes targeting the SARS-CoV-2 N gene. The reference standard was serially diluted by 1o-fold from 1 × 10^10^ copies to 1 × 10^4^ copies. PCR amplification cycle was 50°C, 15 min; 95°C, 3 min; 95°C, 15 s; 60°C, 45 s + Plate Read, 50 cycles, and data were processed by Bio-Rad CFX Manager software.

### Western blotting

Cells were lysed on ice using RIPA lysis buffer (50 mM Tris-HCl, 150 mM NaCl, 1 mM EDTA, 1% NP-40, 0.25% sodium deoxycholate, and adjusted to pH 7.4) supplemented with protease inhibitor cocktail (Roche). Following 30 min at 4°C with periodic vortex, lysates were centrifuged at 12,000 × *g* for 15 min at 4°C to remove cell debris. The protein-containing supernatant was collected. Protein concentrations were determined using a BCA Assay Kit (Thermo Fisher Scientific, 23225). Equal protein amounts (20–50 µg, as determined by BCA assay) were resolved by SDS-PAGE and transferred onto nitrocellulose membranes (Pall, 66485). Membranes were blocked with 5% non-fat milk in 1× phosphate buffered saline with 0.1% Tween-20 (PBST) for 1 h at room temperature. Subsequently, the membranes were incubated with primary antibodies overnight at 4°C. After washing with PBST, the membranes were incubated with IRDye Fluor 680/800-labeled secondary antibody for 1 h at room temperature. Protein bands were visualized using an Odyssey infrared imaging system (Li-COR, Odyssey).

### Time-of-drug-addition assay

The time-of-drug-addition experiment was used as previously described (52). In brief, HeLa-ACE2 cells were seeded in 24-well plates at a density of 1 × 10^5^ cells per well. To evaluate the effects of treatment at different stages of the viral life cycle, three experimental groups were established: full-time, entry, and post-entry. For the full-time assay, cells were pretreated with 4 µM UNC0638 for 1 h, and the treatment was maintained throughout the entire infection period. For entry assay, cells were pretreated with 4 µM UNC0638 for 1 h prior to viral inoculation, and the treatment was only maintained during the viral adsorption phase. For post-entry assay, UNC0638 was added 1 h after viral incubation, and the treatment was maintained for the remainder of the infection period.

### SARS-CoV-2 S pseudovirions production and infection assay

Pseudovirions were generated as previously described (21, 45). Briefly, PSPAX2 (packaging plasmid), plenti-GFP (a reporter plasmid), and plasmids encoding either SARS-CoV-2 S or VSV glycoprotein were co-transfected into HEK 293T cells using polyetherimide (Sigma-Aldrich, 408727). After 48 h, the supernatants containing pseudovirions were harvested, centrifuged at 2,000 × *g* for 10 min to remove cell debris, aliquoted, and stored at −80°C.

For infection assays, HeLa-ACE2 and A549-ACE2 cells were seeded in 96-well plates at a density of 1 × 10^4^ cells per well. The next day, cells were pretreated with UNC0638, BIX01294, and UNC0642 for 1 h prior to infection. Cells were then infected with pseudovirions in the presence of polybrene (Solarbio Life Science, H8761) to enhance viral entry. After 24 h, the culture media were replaced with fresh media to remove unbound or extracellular pseudovirions. At approximately 24 h post-infection, cells were lysed with 40 µL passive lysis buffer (Promega, E1941) at 4°C for 30 min. Luminescence was measured using the Bright-Lite Luciferase Assay System (Vazyme, DD1204-02) and quantified with a Modulus II microplate reader (Turner Biosystems, Sunnyvale, CA, USA).

### Viral binding and internalization assay

Viral binding and internalization assays were performed as previously described (20, 21). For the binding assay, HeLa-ACE2 cells were pretreated with UNC0638 for 1 h, followed by incubation with SARS-CoV-2 (MOI = 5) at 4°C for 1 h to allow viral attachment. After incubation, cells were washed three times with pre-chilled Dulbecco’s phosphate-buffered saline (DPBS) to remove unbound viruses.

For the internalization assay, HeLa-ACE2 cells were similarly pretreated with UNC0638 for 1 h and then incubated with SARS-CoV-2 (MOI = 5) at 4°C for 1 h. After washing with cold DPBS to remove unbound virus, cells were transferred to 37°C for 30 min to allow viral internalization. To remove the uninternalized virus, cells were treated with 0.25% trypsin for 10 min at 4°C. The relative intracellular viral RNA was quantified by RT-PCR and normalized to GAPDH.

### Cell-cell membrane fusion

HEK 293T cells were co-transfected with plasmids encoding the SARS-CoV-2 S glycoprotein and EGFP. After 24 h of transfection, the S-expressing cells were overlaid onto a 90% confluent monolayer of HeLa-ACE2 or HEK 293T cells at a ratio of three ACE2-expressing cells to one S-expressing cell. Cells were treated with UNC0638 and incubated for 12 h at 37°C. Images of syncytia formation were captured with a fluorescence microscope (ZEISS, ApoTome 2).

### S protein cleavage assay

An S protein cleavage was performed following a previously described protocol (20). HeLa-ACE2 cells were seeded at a density of 8 × 10^4^ cells per well in 24-well plates. The following day, cells were pretreated with UNC0638 for 1 h, followed by infection with 500 µL of pseudovirions at 4°C for 1 h to allow viral binding. After incubation, cells were washed three times with cold DPBS to remove unbound virus and then incubated with pre-warmed medium at 37°C for the indicated time points to facilitate viral entry and S processing. Cell lysates were collected at indicated time points using RIPA lysis buffer. Cells treated with bafilomycin A1 served as a negative control for S cleavage. The cleavage of S protein was analyzed by western blot.

### EGF-mediated EGFR degradation assays

HeLa-ACE2 Cells were seeded at 1 × 10^5^ cells per well in 24-well plates. The following day, cells were pretreated with UNC0638 or bafilomycin A1 for 1 h. After treatment, EGF was added to the cells at a final concentration of 40 ng/mL. At different time points, cells were lysed with RIPA lysis buffer. The degradation of EGFR was assessed by western blot.

### Statistical analysis

All data are presented as mean ± standard deviation. Statistical analysis was performed using an independent samples *t*-test with Graph Prism software. Significance levels are indicated as follows: ***P < 0.001, **P < 0.01, *P < 0.05, ns *P* > 0.05.

## Data Availability

All data from this study are included in the paper and are available from the corresponding author upon reasonable request.
